# Contemporary evidence: baseline data from the D2B Alliance

**DOI:** 10.1186/1756-0500-1-23

**Published:** 2008-06-11

**Authors:** Elizabeth H Bradley, Brahmajee K Nallamothu, Amy F Stern, Jason R Byrd, Emily J Cherlin, Yongfei Wang, Christina Yuan, Ingrid Nembhard, John E Brush, Harlan M Krumholz

**Affiliations:** 1 Section of Health Policy and Administration, School of Public Health, Yale University School of Medicine, New Haven, Connecticut, USA; 2 Robert Wood Johnson Clinical Scholars Program, Department of Medicine, Yale University School of Medicine, New Haven, Connecticut, USA; 3 Health Services Research and Development Center of Excellence, Ann Arbor VA Medical Center, and the Division of Cardiovascular Disease, Department of Internal Medicine, University of Michigan Medical School, Ann Arbor, Michigan, USA; 4 American College of Cardiology, Washington, District of Columbia, USA; 5 Section of Cardiovascular Medicine, Department of Medicine, Yale University School of Medicine, New Haven, Connecticut, USA; 6 Sentara Cardiovascular Research Institute, Norfolk, Virginia, USA; 7 Center for Outcomes Research and Evaluation, Yale-New Haven Hospital, New Haven, Connecticut, USA

## Abstract

**Background:**

Less than half of U.S. hospitals meet guidelines for prompt treatment of ST-segment elevation myocardial infarction (STEMI). The Door-to-Balloon (D2B) Alliance is a collaborative effort of more than 900 hospitals committed to implementing a set of evidence-based strategies for reducing D2B time. This study presents data on (1) the prevalence of evidence-based strategies in U.S. hospitals that participated in the D2B Alliance and (2) identifies key hospital characteristics associated with their use.

**Methods:**

We conducted a cross-sectional study of U.S. hospitals that joined the D2B Alliance through a Web-based survey about their current practices for patients with STEMI who received primary percutaneous coronary intervention (PCI). We used multivariate logistic regression to identify hospital characteristics associated with use of each strategy.

**Results:**

Of the 915 U.S. hospitals enrolled in the D2B Alliance as of June 2007, 797 (87%) completed the survey. Only 30.4% of responding hospitals reported employing at least 4 of the 5 key strategies (emergency medicine activates catheterization laboratory, single-call activation, expectation that catheterization team is available in the laboratory within 20–30 minutes after page, prompt data feedback on D2B times, use of pre-hospital electrocardiograms to activate the laboratory while the patient is en route to the hospital); 9.3% employed none of the strategies. There was no clear pattern of correlation between hospital characteristics and reported strategies.

**Conclusion:**

As of 2007, many hospitals had implemented few of the key strategies to reduce D2B time, suggesting substantial opportunity to improve care for patients with STEMI.

## Background

Despite remarkable improvement in many quality indicators for acute myocardial infarction [1,2], national performance in hospital door-to-balloon (D2B) times (i.e., time between hospital arrival and angioplasty balloon inflation) for patients with ST-segment elevation myocardial infarction (STEMI) lags. As of 2005, less than half of U.S. hospitals had median D2B times of 90 minutes or less [3], the American College of Cardiology/American Heart Association guideline for treatment of patients with STEMI. Previous studies of hospitals that consistently meet this guideline reveal several organizational strategies that are associated with faster D2B times [4-16]. In our investigation of hospital practices in 2005 [3], only a minority of hospitals reported using these time-saving strategies, but more contemporary information is not available in the medical literature.

Having contemporary data on the prevalence of these strategies is important for identifying the degree to which greater efforts in improvement are needed in this area. If most hospitals have adopted the strategies identified in 2005 [3] as important, then further initiatives such as the D2B Alliance [17] – a quality improvement effort of more than 900 hospitals committed to implementing a set of evidence-based strategies for reducing D2B time – and other national collaborative efforts may be less important. In contrast, if the prevalence of the strategies remains modest, it is essential to support continued efforts to improve organizational practices in this area.

To provide the most contemporary data on hospital strategies available, we used a baseline cross-sectional survey of hospitals completed at the time of their initial enrollment (November 2006–June 2007) with the D2B Alliance. In this report, we present data on the prevalence of these strategies and identify key hospital characteristics associated with their use.

## Methods

### Study design and sample

Between November 2006 and January 2007, we conducted a cross-sectional study of all U.S. hospitals that joined the D2B Alliance as of June 2007 (n = 915), all of which perform primary PCI for patients with STEMI. Hospitals completed a Web-based survey, which has been previously described [3], about their current practices in caring for patients with STEMI receiving PCI. Of the 915 U.S. hospitals enrolled with the D2B Alliance, 797 (87%) completed the survey. Respondents were more likely than non-respondents to be larger, nonprofit, and teaching hospitals (P-values < 0.05). All research procedures were approved by the Human Investigation Committee at Yale University School of Medicine.

### Data and Measures

#### Outcomes variables

The outcomes were the reported presence of key strategies recommended by the D2B Alliance to improve D2B time. The key strategies included emergency medicine activation of the catheterization laboratory, activation with a single call, expectation that the catheterization team is in the laboratory within 20–30 minutes of page, prompt data feedback on D2B times provided for emergency department and catheterization laboratory staff, and use of a pre-hospital electrocardiogram to activate the catheterization laboratory while the patient is still en route to the hospital. In addition, we created a summary measure of the total number of these key strategies that were implemented. The presence of strategies was measured with the Web-based survey, completed by the primary hospital contact person for the D2B Alliance campaign. The respondent was asked to consult with a variety of staff at the hospital in order to reflect the hospital perspective on strategies used for patients receiving primary PCI. The respondent who submitted the survey reflected the diversity of staff leading D2B improvement efforts, with roles as directors of quality improvement, nurse managers, catheterization laboratory directors, emergency department directors, emergency medicine physicians, cardiologists, and a range of clinical managers.

#### Independent variables

Independent variables included hospital characteristics (e.g., number of staffed beds, ownership type, geographic location in 9 Census regions, and teaching status where teaching was defined as having an Accreditation Commission for Graduate Medical Education residency program). All hospital characteristics were obtained from the American Hospital Association Annual Survey of Hospitals.

### Data analysis

We used standard frequency analyses to describe the hospital sample and to report the prevalence of each D2B Alliance recommended strategy, as well as the mean number of reported strategies. To examine if some hospital types were more likely to implement certain strategies, we used logistic regression models to estimate the adjusted associations between each strategy (i.e., the outcomes) and key hospital characteristics as independent variables (i.e., number of staffed beds, ownership type, geographic location, and teaching status). We used a multiple linear regression model to examine the adjusted association between the number of strategies implemented and all hospital characteristics. All models included all hospital characteristics as independent variables. All data analyses were performed using SAS software, version 9.1 (SAS Institute).

## Results

**Figure 1:**
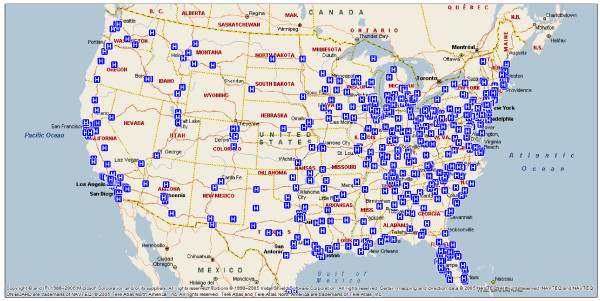
Map of D2B Alliance member hospitals as of June 2007.

Among the 797 respondent hospitals (Table 1), the mean number of staffed beds was 376 (standard deviation (SD) 238.6), and nearly three-quarters of the hospitals were non-profit. The hospitals were diverse in geographic location (Figure 1), and just less than half were teaching hospitals.

**Table 1:** Hospitals that enrolled in the D2B Alliance (n = 797)

**Characteristics**	**N**	**%**
**Number of beds**		
<300	340	43.9
300 – 600	335	43.2
>600	100	12.9
Unknown	22	---
Mean (SD)	376	238.6
**Ownership type**		
Nonprofit	573	73.9
For-profit	124	16.0
Governmental	78	10.1
Unknown	22	---
**Census region**		
New England	29	3.6
Mid-Atlantic	92	11.8
South Atlantic	144	18.1
East North Central	169	20.2
East South Central	68	8.5
West North Central	55	6.9
West South Central	101	12.7
Mountain	55	6.9
Pacific	82	10.5
**Teaching status**		
Non-teaching hospital	416	53.7
Teaching hospital	359	46.3
Unknown	22	---

D2B, door-to-balloon; SD, standard deviation

**Figure 2:**
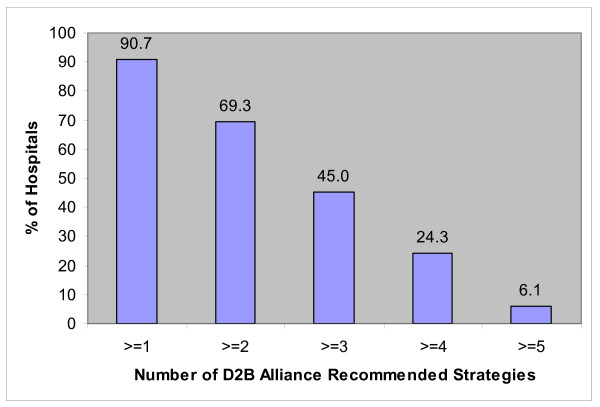
Number of D2B recommended strategies reported by hospitals.

The reported prevalence rates of key strategies recommended by the D2B Alliance are shown in Table 2. The most prevalent of the key strategies was the hospital's expectation that the catheterization team be available in the laboratory within 20–30 minutes of being paged, with more than 80% of hospitals reporting that this expectation was in place. Approximately half of hospitals report that emergency medicine physicians activate the catheterization laboratory. Similarly, about half report that there is prompt (within 1 week) data feedback about D2B times. The less commonly reported strategies (reported by less than one third of hospitals) were activating the catheterization laboratory with a single-call system and using pre-hospital electrocardiograms to activate the catheterization laboratory while the patient is still en route to the hospital. Only 6.1% of hospitals had all 5 strategies; a total of 30.4% of hospitals reported having at least 4 of the strategies, while 9.3% reported having none of the strategies (Figure 2).

**Table 2:** Prevalence of reported D2B Alliance strategies (n = 797)*

**Recommended Strategies**	**Prevalence n/N (%)**	**95% CI**
**Emergency medicine physician activates the catheterization laboratory**		
On day shifts	394/763 (51.6)	48.1, 55.2
On night and weekend shifts	455/763 (59.6)	56.1, 63.1
On *both *day and night/weekend shifts	391/761 (51.4)	47.8, 55.0
**Catheterization laboratory is activated through a single-call system using page operator**	229/761 (30.1)	26.8, 33.4

**Catheterization team is expected to be in the catheterization laboratory within 20–30 minutes of page**		
Catheterization laboratory nurses and technicians	661/749 (88.3)	85.9, 90.6
Interventional cardiologist	629/746 (84.3)	81.7, 86.9
Catheterization laboratory nurses, technicians, *and *interventionalist	607/745 (81.5)	78.7, 84.3
**Prompt data feedback about D2B times is provided to emergency department and catheterization laboratory staff (within 1 week)**	409/771 (53.1)	48.5, 56.6

**Activate catheterization laboratory based on pre-hospital electrocardiogram while patient is still en route to hospital**	241/747 (32.3)	28.9, 35.6

* Denominator for all percentages is total non-missing responses to relevant item.

CI, confidence interval

There was no clear pattern of correlation between hospital characteristics and reported strategies (Table 3). Larger hospitals were significantly more likely to have implemented 3 of the strategies (emergency medicine activation, single-call activation, and activation based on pre-hospital electrocardiogram while the patient is still en route) in multivariable analysis, but size was not associated with the other strategies. Patterns in geographic location and in teaching status were not consistent across strategies, and ownership was not significantly associated with any strategy.

**Table 3:** Reported strategies by type of hospital

**Hospital Characteristics**	**EM activates cath lab (%)**	**Single call activation (%)**	**Cath team available 20–30 mins (%)**	**Data feedback (<1 week) (%)**	**Activate while pt en route (%)**
**Staffed beds**					
<300 (reference)	46.9	25.7	82.0	54.1	26.9
300 – 600	53.4	28.1	80.4	51.5	34.8*
>600	62.6*	50.5*	83.7	58.5	40.6*
**Ownership type**					
Nonprofit (reference)	53.0	31.6	82.3	52.7	32.8
For-profit	49.6	21.8	79.3	58.2	32.8
Governmental	46.6	31.9	79.2	52.1	25.7
**Census region**					
New England (reference)	60.7	48.3	79.3	42.9	24.1
Mid-Atlantic	48.9	33.7	76.7	59.3	27.0
South Atlantic	54.7	33.6	78.8	64.0	33.1
East North Central	52.2	35.0	79.5	45.1	33.3
East South Central	32.8*	18.8*	82.0	41.5	15.9
West North Central	61.1	46.3	96.2*	47.3	30.8
West South Central	47.9	18.6*	86.2	53.5	29.2
Mountain	43.4	24.5	79.3	46.2	32.7
Pacific	61.5	19.2*	81.8	64.2	55.8*
**Teaching status**					
Non-teaching (reference)	49.1	22.5	83.9	55.1	32.7
Teaching	54.9	38.6*	78.8*	51.7	31.5
N	761	761	745	771	747

P-value < 0.05 in fully adjusted logistic regression model

EM, Emergency Medicine

## Discussion

We found that implementation of recommended strategies at the time of enrollment in the D2B Alliance (late 2006 through mid-2007) was limited for many enrolled hospitals, suggesting ample opportunity for improvement among these hospitals. For instance, emergency medicine physician activation of the catheterization laboratory and prompt data feedback about D2B times were being implemented by just about half of hospitals, and a single-call system of activation or use of pre-hospital electrocardiograms while the patient is en route to the hospitals were used by less than one third of hospitals. Given the strong evidence of the impact of these strategies on D2B times and the persistent underutilization of effective strategies, there remains tremendous opportunity to improve quality of care and reduce mortality for patients with STEMI.

The study underscores the need for quality improvement initiatives and the potential impact of the D2B Alliance. Although a previous study demonstrated that many hospitals were not implementing these strategies in 2005, it was not known if there had been substantial adoption since that time. Our study indicates that even among hospitals that were committed to reducing D2B times by joining the D2B Alliance, the prevalence of some key strategies was modest, as recently as early 2007. Future evaluation of the D2B Alliance experience will be necessary to demonstrate if such a collaborative effort can facilitate adoption of these key strategies to reduce D2B times.

The less commonly implemented strategies reflect particular challenges. Single-call systems are difficult without a unified on-call list for interventional cardiologists, an innovation that requires strong commitment and collaboration across potentially competitive clinical groups. Similarly, building the necessary systems of care to coordinate Emergency Medical Services and hospitals so that catheterization laboratories can be activated before the patient arrives at the hospital may require capital equipment, training of emergency medical personnel, and collaboration across service providers not under the control of the hospital. Nevertheless, recent studies have shown that such systems of care with Emergency Medical Services are feasible and effective [12,18,19], suggesting this approach could be a powerful intervention to improve STEMI care.

Importantly, traditional hospital characteristics, such as hospital size and teaching status, are not strongly correlated with reported approaches to reducing D2B time. Rather, hospitals that have been successful in reducing D2B time substantially demonstrate a clear organization-wide goal to reduce D2B time, strong administrative support for improvement efforts, uncompromising clinical leadership to promote improvement, and effective collaboration among departments and disciplines [4]. These data suggest that improvements in D2B time are possible in a variety of types of hospitals regardless of size and teaching status, although organizational commitment and both clinical and administrative leadership are central to effecting needed changes.

Although this study provides the most current data on the implementation of recommended strategies for reducing D2B time, the data should be interpreted in light of their limitations. First, the data are self-reported, although we used an instrument that had been pre-tested and used in previous studies of this nature. Furthermore, we anticipate that self-reporting might bias the prevalence data upwards so that our reported findings may actually overestimate the true use of these strategies. Second, surveys were completed by a single respondent, although researchers were clear with respondents that they should consult with other hospital staff as necessary before reporting the existing processes in place at the hospital. The issue of documenting organizational practices is complex; however our approach was designed to reflect the combined views of key staff involved with treating patients with STEMI. Nevertheless, to the degree respondents knew what was recommended, this limitation may bias the prevalence upwards so that our findings may, again, overestimate the true use of these strategies. Third, although the sample is large and the response rate is high, especially for organizational studies of this type, the data reflect D2B Alliance hospitals rather than a random sample of hospitals that perform PCI. Experience in hospitals that did not choose to enroll in the D2B Alliance may be different. Finally, our study did not examine hospital strategies that might be linked to faster door-to-needle times, as it was beyond the scope of the study.

## Conclusion

In conclusion, this study establishes that key strategies associated with more rapid D2B times are underutilized across the country. Although many hospitals use some of these key strategies, few have adopted them all. Approaches to speeding adoption are varied; participation in inter-organizational learning efforts, such as the D2B Alliance, may be effective in promoting the efficient diffusion of such strategies. However, evaluation of its effectiveness awaits the completion of ongoing and future studies. Our findings highlight the opportunity to improve care and provide baseline data so that the effect of the D2B Alliance can be rigorously evaluated in the future.

## Competing interests

The authors declare that they have no competing interests.

## Authors' contributions

EB designed the study and analysis, and drafted the manuscript. BN designed the survey and analysis and revised the manuscript. AS and JB implemented the survey and revised the manuscript. EC collected and analyzed the survey data. YW analyzed the data and performed the data management. CY collected data. IN revised the manuscript. JB assisted in the study design. HK designed the study and analysis, and revised the manuscript. All authors have read and approved the final manuscript.
